# The association between frailty and survival in patients with pleural disease: a retrospective cohort study

**DOI:** 10.1186/s12890-024-02981-3

**Published:** 2024-04-16

**Authors:** Eleanor Barton, A. Verduri, B. Carter, J. Hughes, J. Hewitt, N. A. Maskell

**Affiliations:** 1 Academic Respiratory Unit, School of Clinical Sciences, University of Bristol, Bristol, UK; 2 Respiratory Unit, Department of Surgical and Medical Sciences, University of Modena and Reggio Emilia, Policlinico Modena, Italy; 3 Department of Biostatistics and Health Informatics, Institute of Psychiatry, Psychology and Neuroscience, Kings College London, London, UK; 4 Department of Population Medicine, Cardiff University, Cardiff, UK

**Keywords:** Pleural disease, Pleural effusion, Malignancy, Frailty, Survival

## Abstract

**Background:**

There are currently no data on the relationship between frailty and mortality in pleural disease. Understanding the relationship between frailty and outcomes is increasingly important for clinicians to guide decisions regarding investigation and management. This study aims to explore the relationship between all-cause mortality and frailty status in patients with pleural disease.

**Methods:**

In this retrospective analysis of a prospectively collected observational cohort study, outpatients presenting to the pleural service at a tertiary centre in Bristol, UK with a radiologically confirmed, undiagnosed pleural effusion underwent comprehensive assessment and were assigned a final diagnosis at 12 months. The modified frailty index (mFI) was calculated and participants classified as frail (mFI ≥ 0.4) or not frail (mFI ≤ 0.2).

**Results:**

676 participants were included from 3rd March 2008 to 29th December 2020. The median time to mortality was 490 days (IQR 161–1595). A positive association was found between 12-month mortality and frailty (aHR = 1.72, 95% CI 1.02–2.76, *p* = 0.025) and age ≥ 80 (aHR = 1.80, 95% CI 1.24–2.62, *p* = 0.002). Subgroup analyses found a stronger association between 12-month mortality and frailty in benign disease (aHR = 4.36, 95% CI 2.17–8.77, *p* < 0.0001) than in all pleural disease. Malignancy irrespective of frailty status was associated with an increase in all-cause mortality (aHR = 10.40, 95% CI 6.01–18.01, *p* < 0.0001).

**Conclusion:**

This is the first study evaluating the relationship between frailty and outcomes in pleural disease. Our data demonstrates a strong association between frailty and 12-month mortality in this cohort. A malignant diagnosis is an independent predictor of 12-month mortality, irrespective of frailty status. Frailty was also strongly associated with 12-month mortality in patients with a benign underlying cause for their pleural disease. This has clinical relevance for pleural physicians; evaluating patients’ frailty status and its impact on mortality can guide clinicians in assessing suitability for invasive investigation and management.

**Trial registration:**

This study is registered with the Health Research Authority (REC reference 08/H0102/11) and the NIHR Portfolio (Study ID 8960).

## Background

As the global population ages, frailty is becoming increasingly common. The term ‘frailty’ describes the cumulative decline in a patient’s physiology over the course of their lifetime, rendering them vulnerable to minor external stressors [1]. Whilst there is a strong correlation between age and frailty, age is at best a surrogate marker for frailty and its effects and cannot be assumed to indicate frailty. Identifying frail patients and understanding the impact that frailty has on clinical outcomes is vital for clinicians.

Frailty can be assessed using a number of validated tools. Two main classifications of frailty, the Phenotype model and the Deficit model, are widely accepted and frequently used in clinical practice. The Phenotype model defines someone as frail if they have three or more of; unintentional weight loss, self-reported exhaustion, weakness (grip strength), slow walking speed, and low physical activity [2]. The deficit model [3] quantifies the cumulative number of a patient’s preselected health deficits and correlates with worse outcomes and higher mortality in nearly every medical and surgical specialty, although remains explored in pleural disease [4–9]. One simplified deficit-based index is the modified 5-item Frailty Index (mFI-5) [10], in which frailty is based on how many specific impactful comorbidities a patient has and whether they are functionally dependent. The mFI provides contrasting results, being reported as a valuable preoperative mortality risk tool in emergency [6] and elective [11, 12] surgery and as poor prognostic instrument in predicting mortality following COVID-19 in older people [7]. 

Pleural disease is common, particularly amongst older people; 1.5 million people develop a pleural effusion annually in the US [13], with 50% attributable to either malignancy or cardiac failure [14] whilst a further 300,000 parapneumonic effusions are reported annually [13], all of which are common conditions in old age. Frailty has been recognised as a negative prognostic factor in any cancer therapy. In thoracic oncology, several studies showed that patients with lung cancer living with frailty have worst survival, irrespective of age and comorbidities [15]. There are currently limited data available on frailty in pulmonary disease; most existing studies explore the relationships between frailty and chronic obstructive pulmonary disease (COPD) and interstitial lung disease (ILD) [9, 16]. 

There are currently no data available on the association between frailty and patient outcomes in malignant and benign pleural disease. Understanding the relationship between frailty and outcomes, including mortality, in this cohort of patients with a high incidence of malignancy is increasingly important when assessing who may be suitable for invasive investigation and management, and in whom clinicians’ priority ought to be prioritising quality of life.

In this study, we hypothesise that frailty, as identified using the modified Frailty Index, in patients with pleural disease is associated with increased mortality. This is the first study in the literature evaluating the association between frailty and pleural disease.

## Methods

This manuscript follows the STROBE statement for reporting of cohort studies.

### Study design

We conducted a retrospective analysis of prospectively collected data from a cohort from 3rd March 2008 to 31st December 2020, by enrolling consecutive adult patients presenting to the tertiary pleural service at North Bristol NHS Trust, UK, with a radiologically confirmed, undiagnosed pleural effusion or pleural thickening into this observational study (IRAS ethics number 08/H0102/11). Patients were referred to the tertiary pleural service from multiple sources, including GP practices, the emergency department, the acute medical unit, inpatient wards and secondary care at other local hospitals. Patients over the age of 18 who were recruited in the outpatient setting were included in this analysis, due to there being more complete data for outpatients than inpatients. All patients underwent comprehensive clinical, radiological and biochemical assessment at enrolment and were assigned a diagnosis by two independent respiratory specialists after 12 months follow up. Mortality data was collected as part of this retrospective analysis for participants still alive at their original 12-month review. Full study details can be found within the previously published study protocol [17]. 

### Variables

Descriptive data to characterize the clinical cohort were collected from the participants’ electronic patient record at participant enrolment:

- Age
- Sex
- Date of presentation
- Asbestos exposure
- Performance status assessment (WHO performance status and Karnofsky performance status) and assessment of functional dependence
- Co-morbidities– specifically diagnoses of Chronic Obstructive Pulmonary Disease (COPD), treated hypertension, diabetes and chronic heart failure at presentation.

#### Performance status and functional dependence assessment

Assessment of the performance status (PS) is an important tool for clinicians to evaluate functional status of patients. The WHO PS score was used in this study, with a threshold score of 3 indicating functional dependence.

#### Primary exposure of frailty

Frailty was assessed using the modified frailty index (mFI). The mFI includes five items: chronic heart failure (CHF); Chronic Obstructive Pulmonary Disease (COPD); diabetes mellitus (DM); treated hypertension (HTN); and functional dependence as the component deficits. The mFI ranges between 0 and 1, with each contributing domain assigned a score of 0.2.

For analyses, mFI was categorised as not frail (mFI ≤ 0.2) and frail (mFI ≥ 0.4). CHF, COPD, DM, and treated HTN were identified using electronic clinical records and hand-searching medical records, where available.

#### Sample analysis

Pleural fluid was sent for biochemical, microbiological and cytological analysis as clinically indicated. Biopsies were obtained if clinically indicated to confirm the diagnosis and identify the histological subtype in cases of suspected malignant pleural effusions. Biopsies were obtained either percutaneously under radiological guidance, by local anaesthetic thoracoscopy or by video assisted thoracoscopic surgery (VATS). Outcomes of these investigations were recorded in the database and used to assign a diagnosis at 12 months.

### Outcomes

#### Primary outcome

The primary outcome was time to all-cause mortality from date of presentation. All-cause mortality was collected one year from enrolment.

#### Secondary outcome

The secondary outcome was time to all-cause mortality from date of study enrolment in malignant and benign pleural disease.

### Data analysis

The analysis plan was authored by a statistician fully blinded to the outcome data (BC) following King’s College London Clinical Trials Unit (KCTU) standard operating procedures on drafting a Statistical Analysis Plan (SAP).

#### Primary analysis

The time to all-cause mortality was assessed visually using a Kaplan-Meier plot with associated at-risk table and log-rank test. All-cause mortality was analysed using an adjusted multivariable Cox baseline proportional hazards regression, adjusting for: age at diagnosis (Under 65, 65 to 79, 80 or older); underlying aetiology; suspected asbestos exposure status, performance status, malignancy status and frailty. Underlying aetiology was classified into malignant disease, benign effusion related to organ failure (including heart, liver and renal failure), inflammatory effusions (including, but not limited, to those related to connective tissue disease, benign asbestos related pleural effusions and post-cardiac surgery effusions), infective effusions and other. We present the crude hazard ratio (HR) and adjusted HR (aHR) with associated 95% confidence intervals and p-values. The baseline proportional hazard assumption was assessed visually using log-log plots.

#### Subgroup analyses

Subgroup analyses for age group, final diagnosis, and malignancy status were performed.

## Results

**Fig. 1:**
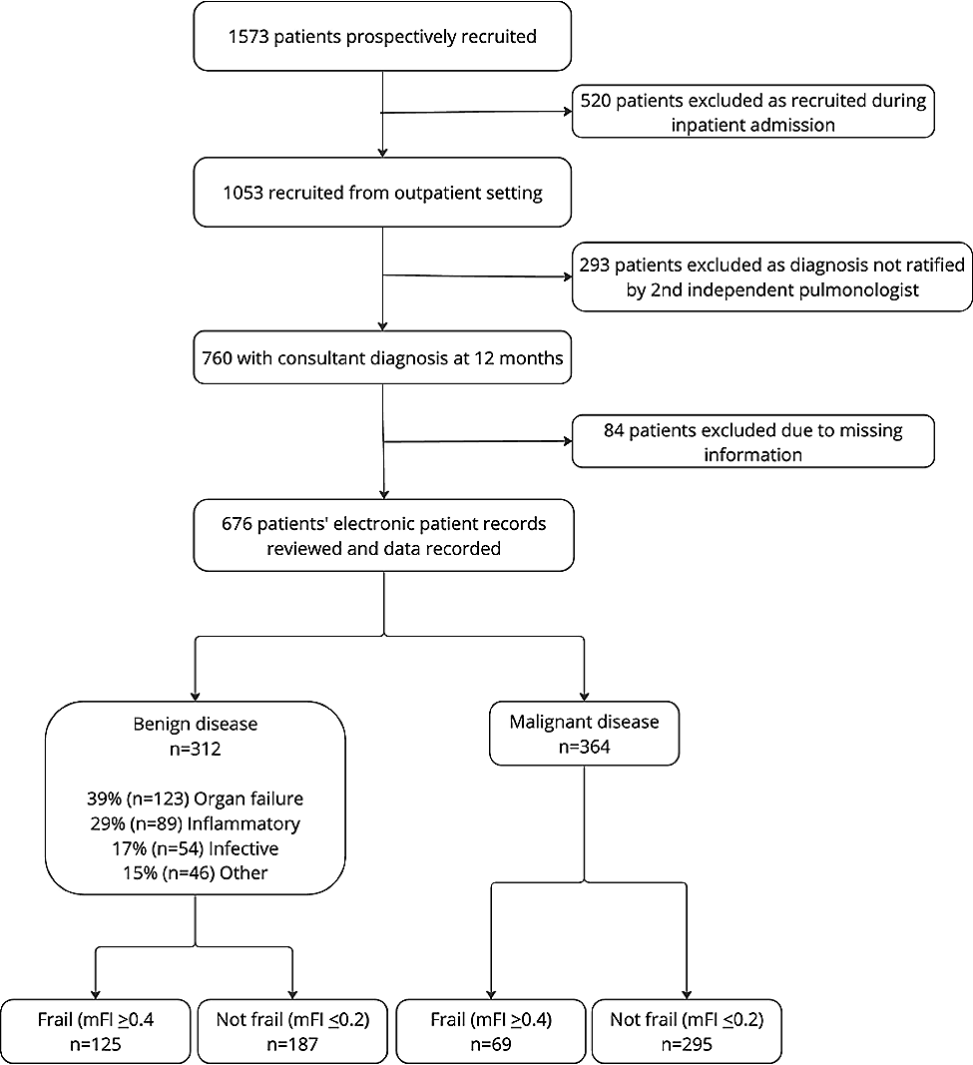
Consort diagram showing participant recruitment, exclusion and analysis

Data were collected for a total of 1573 patients. 520 were excluded as they were recruited during an inpatient stay and a further 293 patients excluded as they had yet to have a second independent pulmonologist ratify their diagnosis for the purposes of the study. Of the remaining 760 outpatients, 84 participants were excluded as co-morbidities or performance status were missing (not recorded). A total of 676 participants were included in the analysis. (Fig. 1)

Baseline characteristics and mortality data at 12 months for the cohort are shown in Table 1. Ages ranged from 23 to 97, with a mean age = 70.7 (12.5). 67% were male (*n* = 453). 54% (*n* = 364) of effusions were secondary to malignancy, the primary site of which is summarized in Table 2 and 18% (*n* = 123) were benign effusions related to organ failure, 13% (*n* = 89) were inflammatory and 8% (*n* = 54) were infective in origin. The remaining 7% (*n* = 46) were classified as “other” and included diffuse pleural thickening, post-surgical effusions, post-traumatic effusions, chylothoraces, effusions related to pulmonary emboli, drug-induced effusions and effusions of unknown origin. The prevalence of frailty in this cohort was 29% (*n* = 194). 24% (*n* = 163) of participants were assessed as being functionally dependent. 17% (*n* = 115) were found to have diabetes, 33% (*n* = 221) treated hypertension, 7% (*n* = 50) COPD and 20% (*n* = 134) chronic heart failure.

**Fig. 2:**
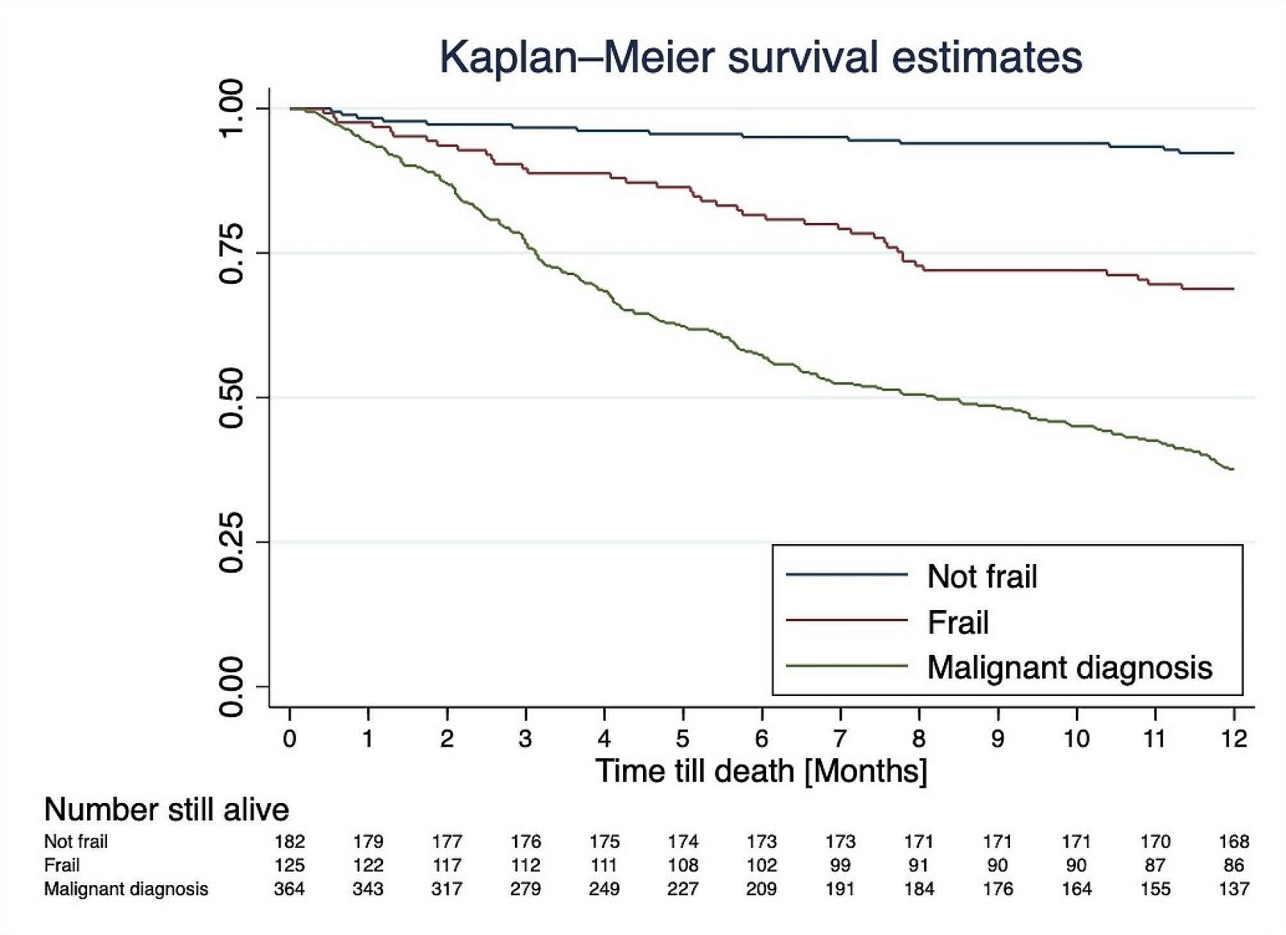
Kaplan-Meier survival curve showing 12-month mortality of patients with pleural disease depending on frailty status or malignant diagnosis.

The median time to mortality was 490 days (IQR 161–1595). The one-year mortality rates for those not frail, frail and with a diagnosis of malignancy were 0.10, 0.30 and 0.62 respectively (Fig. 2). There was an association between mortality and frailty (log-rank *p* < 0.001).

In the primary analysis an increase in mortality was found for those living with frailty at enrolment (aHR = 1.72, 95% CI 1.07–2.76, *p* = 0.025, Table 3 ), which was increased when a patient was living with a malignant diagnosis (aHR = 2.85, 95% CI 1.75–4.57, p = < 0.0001). Similar findings were reported in the crude analysis. Increasing age and WHO PS (summarized in Table 3) and functional dependence (HR = 2.79, 95% CI 2.18–3.57, p = < 0.0001) were also positively associated with shorter time to all-cause mortality. There was no association found between gender or comorbidities and time to all-cause mortality.

Subgroup analyses evaluated the impact of frailty on mortality at 12 months in patients with benign disease; frailty (mFI ≥ 0.4) conferred an increased risk of mortality within 12 months of presentation (aHR = 4.36, 95% CI 2.17–8.77, *p* < 0.0001) compared to all frail patients with pleural disease. A malignant diagnosis, irrespective of frailty status, was strongly associated with mortality within 12 months (aHR = 10.40, 95% CI 6.01–18.01, *p* < 0.0001) compared to non-frail patients with benign disease. (Fig. 2; Table 4)

**Table 1:** Mortality at 12 months by baseline characteristic

Baseline Clinical Characteristics		1-year mortality		N = XXX (%)
		Dead n (row percentage%)	Alive N (row percentage %)	
Gender	Male	177 (39%)	276 (61%)	453 (67%)
	Female	105 (47%)	118 (53%)	223 (33%)
Age	≤ 64	57 (31%)	126 (69%)	183 (27%)
	65–79	119 (38%)	198 (62%)	317 (47%)
	≥ 80	106 (60%)	70 (40%)	176 (26%)
Asbestos exposure	Unexposed	200 (45%)	246 (55%)	446 (66%)
	Suspected exposed	82 (36%)	148 (64%)	230 (34%)
Diagnostic category	Benign effusion related to organ failure	37 (30%)	86 (70%)	123 (18%)
	Malignant effusion	227 (62%)	137 (38%)	364 (54%)
	Infective aetiology	5 (9%)	49 (91%)	54 (8%)
	Inflammatory aetiology	6 (6%)	83 (94%)	89 (26%)
	Other	7 (15%)	39 (85%)	46 (7%)
Frailty	Not frail (mFI ≤ 0.2)	192 (40%)	283 (60%)	475 (71%)
	Frail (mFI ≥ 0.4)	88 (45%)	106 (55%)	194 (29%)
WHO PS	0	21 (18%)	95 (82%)	116 (18%)
	1	106 (37%)	182 (63%)	288 (44%)
	2	88 (51%)	83 (49%)	171 (26%)
	3	50 (70%)	21 (30%)	71 (11%)
	4	4 (100%)	0 (0%)	4 (1%)
Functional dependence		107 (66%)	56 (34%)	163 (24%)
DM		43 (37%)	72 (63%)	115 (17%)
Treated HTN		84 (38%)	137 (62%)	221 (33%)
COPD		19 (38%)	31 (62%)	50 (7%)
CHF		42 (31%)	92 (69%)	134 (20%)

**Table 2:** Causes of malignant pleural effusions

Causes	Patients, N (%)
Mesothelioma	117 (32%)
Lung cancer	102 (28%)
Breast cancer	51 (14%)
Haematological malignancy	24 (7%)
Gynaecological malignancy	22 (6%)
Cancer of unknown primary	15 (4%)
Gastrointestinal and hepatobiliary cancer	11 (3%)
Urological malignancy	8 (2%)
Melanoma	4 (1%)
Sarcoma	3 (1%)
Head/Neck cancer	3 (1%)
Other (including thymic, carcinoid, neuroendocrine and primary peritoneal malignancies)	4 (1%)

**Table 3:** Crude and adjusted hazard ratios for mortality at 12 months

Characteristic		Crude HR (95% CI)	p value	Adjusted HR (95% CI)	p value
Gender	Female	Reference			
	Male	0.74 (0.58–0.94)	0.013	1.12 (0.85–1.47)	0.438
Age	≤ 64	Reference			
	65–79	1.24 (0.90–1.70)	0.18	1.14 (0.81–1.60)	0.467
	≥ 80	2.38 (1.72–3.29)	< 0.0001	1.72 (1.17–2.52)	0.006
Asbestos exposure	No	Reference			
	Yes	0.71 (0.55–0.92)	0.009	0.62 (0.46–0.84)	0.002
Diagnostic category	Benign effusion due to organ disease	Reference			
	Malignant effusion	2.71 (1.91–3.84)	< 0.0001	2.85 (1.75–4.57)	< 0.0001
	Infective	0.27 (0.11–0.68)	0.006	0.32 (0.12–0.85)	0.023
	Inflammatory	0.20 (0.08–0.47)	< 0.0001	0.27 (0.11–0.69)	0.006
	Other	0.47 (0.21–1.04)	0.064	0.50 (0.21–1.19)	0.118
Frailty	Not frail (mFI ≤ 0.2)	Reference			
	Frail (mFI ≥ 0.4)	1.13 (0.88–1.46)	0.33	1.72 (0.107–2.76)	0.029
WHO status	0	Reference			
	1	2.29 (1.44–3.66)	< 0.0001	1.96 (1.21–3.17)	0.006
	2	3.73 (2.32–6.01)	< 0.0001	2.57 (1.54–4.29)	< 0.0001
	3	6.59 (3.96–10.99)	< 0.0001	4.71 (2.69–8.25)	< 0.0001
	4	81.45 (26.73-248.16)	< 0.0001	53.87 (16.85-172.17)	< 0.0001
DM		0.84 (0.61–1.16)	0.297	0.75 (0.49–1.14)	0.173
Treated HTN		0.81 (0.63–1.05)	0.112	0.66 (0.47–0.93)	0.015
COPD		0.86 (0.54–1.37)	0.517	0.76 (0.45–1.27)	0.294
CHF		0.65 (0.47–0.90)	0.010	0.53 (0.32–0.89)	0.016

**Table 4:** Subgroup analysis– impact of frailty on mortality in benign disease and those with malignant diagnoses

Characteristic		Crude HR (95% CI)	p value	Adjusted HR (95% CI)	p value
Frailty	Not frail (mFI ≤ 0.2)	Reference			
	Frail (mFI ≥ 0.4)	4.65 (2.53–8.57)	< 0.0001	4.36 (2.17–8.77)	< 0.0001
	Malignant diagnosis irrespective of frailty status	12.04 (7.01–20.68)	< 0.0001	10.40 (6.01–18.01)	< 0.0001

## Discussion

This retrospective observational cohort study is the first to explore the relationship between frailty and pleural disease and demonstrates an association between 12-month mortality and frailty (mFI ≥ 0.4) in this cohort. Whilst Respiratory physicians are well accustomed to recording performance status, and both WHO and Karnofsky performance status are known to be predictors of mortality in patients with malignant effusions [18–21], frailty indices are often poorly reported.

The study population was broadly comparable to other studies in similar cohorts [14, 19–21] although the male-to-female ratio and median age were slightly higher. Rates of co-morbidities used to calculate the mFI (COPD, CHF, DM and treated HTN) were higher in our study cohort than the general population of the UK [22–25], likely reflecting the co-morbid nature of patients with pleural disease and the high prevalence of frailty in this cohort.

These data show an association between mortality within 12 months of presentation with pleural disease and both frailty status (mFI ≥ 0.4) and age ≥ 80; an association well recognized in clinical practice. This association was not as strong as expected; we hypothesise that this may relate to the high rates of malignant effusions within our cohort. A malignant pleural effusion is, by definition, metastatic disease, which usually conveys a poor prognosis irrespective of performance or frailty status. In our cohort, non-frail patients with a malignant diagnosis and an inherently poor prognosis likely confound the effect of frailty on 12-month mortality.

In our subgroup analysis, the stronger association between frailty and 12-month mortality in benign disease supports our experience in clinical practice and studies exploring the effect of frailty in other diseases and settings, including surgery and trauma [26, 27]. That malignancy irrespective of frailty status confers the highest risk of 12-month mortality is also not surprising. Other studies have demonstrated that those with a terminal diagnosis, classified in these studies as having a Clinical Frailty Scale (CFS) of 9, have significantly poorer outcomes following trauma, presumably secondary to the poor physiological reserve associated with their underlying disease [26]. The data from our analysis support our hypothesis that a diagnosis of a malignant pleural effusion is an independent predictor of 12-month mortality, irrespective of frailty status.

Understanding the impact of frailty on outcomes in pleural disease is of great clinical value. Patients with effusions frequently have a high symptom burden, but procedures available to manage symptoms and identify the underlying cause of their effusion are invasive and not without risk. The mFI gives clinicians an appreciation of a patient’s frailty status and this study provides valuable insight into how this frailty status is associated with their prognosis. Patients who are frail, and therefore have a poor prognosis, may have different care priorities than a non-frail patient; the focus of their care may centre around maintaining their quality of life and limiting invasive procedures to those which provide the most symptomatic relief with the least risk and fewest hospital attendances, for example by inserting an indwelling pleural catheter (IPC) to manage a patient’s recurrent pleural effusion in the community, rather than repeated ad-hoc drainages or an inpatient admission for a chest drain and talc pleurodesis to reduce their risk of reaccumulation. An improved understanding and awareness of the impact of frailty on patient outcomes would allow clinicians to make pragmatic decisions regarded reducing hospital visits for appointments and scans and may allow patients better access to community services for the frail.

Malignancy is a common cause of pleural disease [14] and treatment with chemotherapy and other systemic anti-cancer therapy can be difficult to tolerate and have significant impact on patients’ quality of life. In mesothelioma in particular, the minimal gain in life expectancy (3 months) [28] needs to be weighed carefully against a patient’s frailty status and the impact that treatment may have on their quality of life.

This study shows that frailty as defined by mFI ≥ 0.4 correlates with 12-month mortality in both benign and malignant disease. This demonstrates that frailty assessment is a tool that can help clinicians make pragmatic, informed decisions regarding management based on prognosis and the patient and clinician’s priorities for their care. It should be noted that it is difficult to tease apart cause and effect in observational studies evaluating frailty and its impact on mortality and we should be wary of prognostic pessimism having too much influence on patient management.

This study has a few limitations; the data is single centre and the nature of this centre as a tertiary referral unit for mesothelioma has led to overrepresentation of mesothelioma patients in the cohort (17% of cohort, 32% of those with malignant effusions), compared to other studies of the same ilk [14, 19]. The study cohort also primarily consisted of outpatients. Data collected for outpatients were more complete than for inpatients, so for this reason, inpatients were excluded from this analysis.

In addition, the spectrum of benign pleural disease is broad, ranging from inflammatory effusions secondary to connective tissue disease or benign asbestos related effusions, which are unlikely to be inherently associated with a poor prognosis, to effusions related to organ failure, which is more likely to be associated with other comorbidities, frailty and poor prognosis. The size of the study cohort precluded subgroup analysis of the relationship between different causes of benign pleural disease and mortality, but this should be explored in future work.

Furthermore, mFI has predominantly been used in surgical settings [6] and data comparing the mFI and the commonly used and more widely validated Clinical Frailty Scale (CFS) [4] has shown it to be less prognostic of outcome [7]. The mFI was chosen in this study for the pragmatic reason that the data required to calculate it were pre-existing within the dataset. Future research may wish to focus on more widely used and validated frailty instruments, such as the CFS, or phenotype models, which could not be calculated in this study due to the data points originally collected. However, it has proven the concept of an association between frailty and pleural disease. Another limitation is that, whilst the largest study of its kind, the absolute number of patients remains small, and this needs to be evaluated in a larger dataset including other pleural diseases, such as pneumothorax.

## Conclusions

This study is the first to show an association between frailty status (mFI ≥ 0.4) and 12-month mortality in pleural disease, irrespective of aetiology. The association is stronger in patients with benign pleural disease, due to the inherently poor prognosis associated with malignant effusions confounding the impact of frailty on outcomes in the whole cohort analysis. This demonstrates the potential for frailty assessment to be used to aid clinicians in making decisions regarding ongoing investigation and management. Further studies are required in larger cohorts including both inpatients and outpatients to further evaluate this.

## Data Availability

The datasets used and/or analysed during the current study are available from the corresponding author on reasonable request.

## Declarations

This study was performed in accordance with the Declaration of Helsinki. This human study was approved by NRES Committee South West– Southmead - approval: 08/H0102/11. All adult participants provided written informed consent to participate in this study. Consent for publication not applicable.

## Abbreviations

aHR: Adjusted hazard ratio
CFS: Clinical frailty scale
CHF: Chronic heart failure
CI: Confidence interval
COPD: Chronic Obstructive Pulmonary Disease
DM: Diabetes mellitus
GP: General Practitioner
HR: Hazard ratio
HTN: Hypertension
ILD: Interstitial Lung Disease
IQR: Interquartile range
KCTU: King’s College London Clinical Trials Unit
mFI: Modified Frailty Index
NHS: National Health Service
PS: Performance status
SAP: Statistical Analysis Plan
STROBE: Strengthening the reporting of observational studies in epidemiology
VATS: Video Assisted Thoracoscopic Surgery
WHO: World Health Organisation
